# Assessing the Role of Fetal Doppler in High-Risk Obstetrics: Evidence From a Comprehensive Study

**DOI:** 10.7759/cureus.68383

**Published:** 2024-09-01

**Authors:** Dipak Kolate, Prashant Suryarao, Nikita Bhattacharjee, Swapnali Sansare

**Affiliations:** 1 Obstetrics and Gynaecology, Dr. D. Y. Patil Medical College, Hospital and Research Centre, Dr. D. Y. Patil Vidyapeeth (Deemed to be University), Pune, IND

**Keywords:** fetal outcome, apgar, umbilical artery, high-risk pregnancy, doppler ultrasonography

## Abstract

Background

Colour Doppler ultrasound is pivotal in modern obstetrics for evaluating maternal and fetal health, especially in high-risk pregnancies. It enhances fetal oxygenation and nutrient delivery assessment, aiding in the early detection of adverse outcomes. However, its effectiveness is influenced by operator skill and the potential for interpretative variability.

Aim

This study aims to assess the utility of Doppler ultrasound in evaluating fetal status in high-risk pregnancies at Dr. D. Y. Patil Medical College, Hospital and Research Centre, Pune, India.

Materials and methods

A hospital-based observational study was conducted from September 2022 to July 2024, including 145 high-risk pregnant women, of whom 120 delivered at the institute. The study included thorough maternal and fetal assessments, with regular Doppler studies starting at 28 weeks of gestation. The frequency of these studies was adjusted according to the changes and severity observed in the indices. Detailed documentation of both maternal and neonatal outcomes was meticulously maintained. Statistical analyses were performed using MS Excel (Microsoft® Corp., Redmond, WA, USA) and RStudio, Version 2023.08.0-daily+170 (RStudio, Inc., Boston, MA, USA), with a significance threshold of p < 0.05.

Results

The majority of participants were aged 21-30 years, with multigravida constituting 63.33%. Pregnancy-induced hypertension (PIH) and preeclampsia were the most common high-risk factors (28.33%). Abnormal umbilical artery (UA) flow patterns were observed in 58.33% of cases. Abnormal UA and middle cerebral artery (MCA) Doppler indices correlated significantly with adverse outcomes. Most deliveries were via caesarean section (82.5%), and 63.33% of neonates required Neonatal Intensive Care Unit (NICU) admission. Neonates with abnormal antenatal Doppler studies had significantly lower APGAR (appearance, pulse, grimace, activity, and respiration) scores and higher NICU admission rates.

Discussion

The study highlights the critical role of Doppler ultrasound in managing high-risk pregnancies, providing essential data for early interventions. Consistent with other studies, abnormal Doppler patterns were significantly associated with adverse neonatal outcomes, necessitating timely caesarean deliveries.

Conclusion

Fetal Doppler ultrasonography is essential for managing high-risk pregnancies, enabling timely therapeutic interventions and improving perinatal outcomes. Despite its limitations, Doppler technology remains invaluable in identifying at-risk foetuses and guiding clinical decisions for optimal pregnancy management.

## Introduction

Colour Doppler is used extensively in modern obstetrics to evaluate maternal and fetal conditions, aiming for optimal management and better pregnancy outcomes. Doppler ultrasound technology relies on the Doppler shift, which describes the change in ultrasound frequency when directed at a moving object, such as a red blood cell [1]. Integrating fetal Doppler findings with biophysical profiles, non-stress tests, and maternal serum biomarkers enhances the assessment and management of high-risk pregnancies, allowing for individualized care plans and improved perinatal outcomes. Fetal Doppler aids in assessing fetal oxygenation and nutrient delivery, detecting fetoplacental perfusion status, and identifying foetuses at risk of adverse outcomes [2]. Despite its advantages, fetal Doppler use has limitations, including variability in interpretation, dependence on operator skill, and the potential for false positives or negatives. Careful consideration of Doppler findings within the overall clinical context is necessary. Nonetheless, when used appropriately, fetal Doppler is a powerful tool in managing high-risk pregnancies, providing detailed information about fetal and placental health, and facilitating timely and effective interventions. 

The goal of this study is to examine how Doppler ultrasonography is used in high-risk pregnancies among the local population that visits our facility.

## Materials and methods

This observational study was carried out at the Department of Obstetrics and Gynaecology at Dr. D. Y. Patil Medical College, Hospital and Research Centre, Pune, India, to assess colour Doppler indices in high-risk pregnancies. Women attending antenatal clinics and labour rooms were screened. We included women with high-risk pregnancies, such as those with preeclampsia or pregnancy-induced hypertension (PIH) in the current pregnancy, fetal growth restriction (FGR), gestational diabetes mellitus (GDM), severe oligohydramnios, and a history of stillbirth, placental abruption, preeclampsia, or FGR in a previous pregnancy. Additionally, women with pre-existing medical conditions, such as diabetes, hypertension, renal diseases, or epilepsy, those planning to deliver at the study centre, and those willing to participate in the study were also included. We excluded women with fetal congenital anomalies or multiple gestations and those whose last menstrual period (LMP) details were unreliable and not confirmed by early ultrasound.

The Institutional Ethics Sub-committee approval (Ref no. IESC/PGS/2022/139) was obtained before the commencement of the study, and eligible women signed informed written consent before the start of the study procedures. Patients attending the OPD and labour room of Dr. D. Y. Patil Medical College, Hospital and Research Centre, who met the inclusion and exclusion criteria, were recruited for the study conducted from September 2022 to July 2024. Our study considered 145 patients with high-risk factors, of whom 120 delivered at our institute. The study included both primigravidae and multipara. After recruitment and obtaining written consent, patients were informed about the study's methodology.

Participating women underwent a comprehensive evaluation, including a detailed history covering menstrual, obstetric, past, family, and personal factors. Gestational age was determined from the LMP and confirmed by an early dating scan. Following a general examination, physical examination, and detailed obstetric examination, relevant investigations were conducted and recorded.

Fetal Doppler studies were performed regularly starting at 28 weeks gestation, using the Samsung HS70 ultrasound machine (Samsung Medison Co., Ltd., Seoul, South Korea), with the frequency adjusted based on the changes and severity of the indices observed. All high-risk pregnancies received regular antenatal fetal monitoring according to the institutional protocol, including colour Doppler ultrasound. Doppler studies of the umbilical artery (UA) and middle cerebral artery (MCA) were performed and documented. Women were monitored until delivery. Delivery details, including mode of delivery (vaginal route or by caesarean section) and neonatal details (birth weight, APGAR (appearance, pulse, grimace, activity, and respiration) scores at five minutes, NICU (Neonatal Intensive Care Unit) admissions, and any neonatal complications such as respiratory distress and neonatal death), were recorded. All maternal and fetal parameters were entered into a data sheet.

Statistical analysis was conducted using MS Excel (Microsoft® Corp., Redmond, WA, USA) and RStudio, Version 2023.08.0-daily+170 (RStudio, Inc., Boston, MA, USA). Categorical variables were analysed using the Chi-square test. For all the tests, a p-value of <0.05 (two-tailed) was considered statistically significant.

## Results

A total of 120 women were included in the study, with an average age of 26.76 years. The largest age group was 21-25 years, representing 35% of the total. This was followed by the 26-30 age group at 30%. Patients aged 31-34 comprised 18%, while those under 20 and over 35 constituted 9% and 5% of the total, respectively. Of the women included in the study, 76 were multigravida.

The most prevalent high-risk pregnancy factors included PIH and preeclampsia, affecting 28.33% of the group, and FGR, affecting 18.3%. Additionally, 2.5% of the group was affected by other factors, such as a history of abruptio placenta and stillbirth (Table 1).

**Table 1:** Distribution based on high-risk pregnancy Data are presented as n(%). PIH: Pregnancy-induced hypertension; FGR: Fetal growth restriction; GDM: Gestational diabetes mellitus

Risk factor	No. of patients	Percentage
FGR	22	18.34
PIH and preeclampsia	34	28.33
Preeclampsia and FGR	18	15
Chronic hypertension	7	5.83
Severe oligohydramnios	12	10
GDM	16	13.34
Overt diabetes	8	6.66
Other	3	2.5

**Figure 1:**
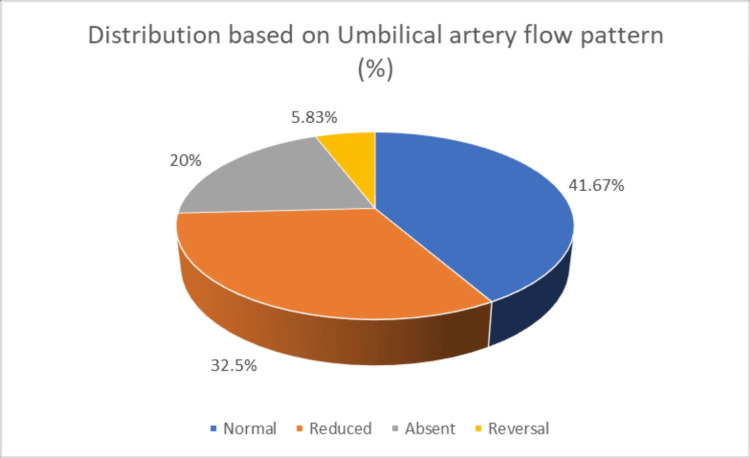
Distribution based on umbilical artery flow pattern

Among the 120 high-risk pregnancies, 41.67% exhibited normal UA flow patterns. However, a significant portion showed abnormal flow patterns: 32.5% had reduced flow, 20% had absent flow, and 5.83% experienced reversed flow (Figure 1).

The abnormal patterns were most prevalent in conditions such as FGR, PIH, preeclampsia, and preeclampsia associated with FGR, highlighting significant placental insufficiency in these high-risk groups (Table 2).

**Table 2:** Distribution based on umbilical artery indices in relation to the high-risk factors considered Data are presented as numbers. PIH: Pregnancy-induced hypertension; FGR: Fetal growth restriction; GDM: Gestational diabetes mellitus

Risk factor	Umbilical artery flow pattern			
	Normal	Reduced	Absent	Reversal
FGR	3	12	6	1
PIH and preeclampsia	11	13	7	3
Preeclampsia and FGR	2	9	4	3
Chronic hypertension	1	4	2	0
Severe oligohydramnios	9	0	3	0
GDM	15	1	0	0
Overt diabetes	7	0	1	0
Other	2	0	1	0

Abnormal UA pulsatility indices (PIs) were observed in 53.33% of patients, while abnormal UA resistive indices (RIs) were observed in 39.16% of patients. In contrast, abnormal MCA indices were less common, with abnormal MCA PI observed in 41.66% of patients and abnormal MCA RI in 27.5% of patients. Normal and abnormal values of UA and MCA indices were found to be statistically significant (p-value <0.001 for both) (Table 3).

**Table 3:** Distribution based on RI and PI of UA and MCA Data are presented as n(%). Test used: Chi-square test; p-value < 0.05 is considered statistically significant. UA: Umbilical artery; MCA: Middle cerebral artery; RI: Resistive index; PI: Pulsatility index

Parameter	UA PI		UA RI		MCA PI		MCA RI		p-value
	Frequency	%	Frequency	%	Frequency	%	Frequency	%	
Normal	56	46.67	73	60.84	70	58.34	87	72.5	<0.001
Abnormal	64	53.33	47	39.16	50	41.66	33	27.5	<0.001

The majority of patients (83/113) had a normal cerebral placental ratio (CPR) (>1), while a smaller subset (30/113) exhibited a reversal (<1) of this ratio.

A total of 99 (82.5%) women underwent lower segment caesarean section (LSCS). In contrast, 21 women (16.67%) had a normal vaginal delivery (NVD) (Table 4).

**Table 4:** Distribution based on mode of delivery Data are presented as n(%). Test used: Chi-square test; p-value < 0.05 is considered statistically significant. LSCS: Lower segment caesarean section; NVD: Normal vaginal delivery

Mode of delivery	Total no. of patients	Percentage	No. of patients with normal Doppler study	No. of patients with abnormal Doppler study	p-value
LSCS	99	82.5	39	60	0.336
NVD	20	16.67	10	10	
Instrumental delivery	1	0.83	1	0	

In the current study, 50% of the infants had a birth weight of <2.5 kg, with a mean birth weight of 2.4 kg. The largest group consisted of infants in the 2.1-2.4 kg range, comprising 34.16% of the population. Following closely, the 2.5-3 kg range included 28.34% of infants. Additionally, 5% of the infants had a birth weight <1.5 kg.

**Figure 2:**
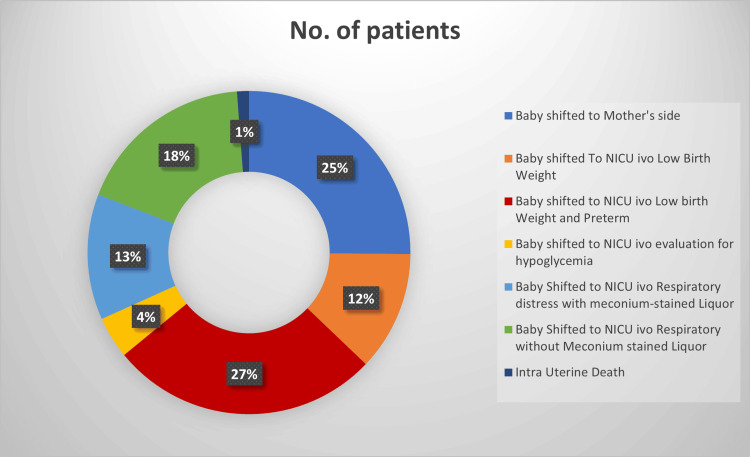
Distribution based on the status of the baby immediately after delivery NICU: Neonatal intensive care unit

While a notable proportion of babies were shifted to the mother's side after delivery, a significant portion required admission to the NICU for various reasons, including low birth weight, preterm birth, hypoglycaemia evaluation, and other health concerns, such as respiratory distress due to meconium-stained liquor (Figure 2). 

Neonates with very low APGAR scores (0-3) at five minutes strongly correlate with abnormal antenatal Doppler studies, indicating that severe Doppler abnormalities were predictive of poor neonatal outcomes (Table 5). Neonates with high APGAR scores (7-10) at five minutes showed a relatively balanced distribution between normal and abnormal Doppler results, indicating that, while normal Doppler studies were somewhat predictive of better outcomes, many neonates with abnormal Doppler results can still achieve high APGAR scores at five minutes with appropriate interventions used in the initial period right after birth. The difference was statistically significant.

**Table 5:** Distribution of neonates with correlation of APGAR at five minutes with antenatal Doppler studies Data are presented as numbers. Test used: Chi-square test; p-value < 0.05 is considered statistically significant. APGAR: Appearance, pulse, grimace, activity, and respiration

APGAR at 5 minutes	No. of neonates	Antenatal normal Doppler	Antenatal abnormal Doppler	p-value
0-3	2	0	2	0.021
4-6	41	11	30	
7-10	77	39	38	

There were two cases of intrauterine death out of 120 newborns, and 76 of them required NICU admission.

Abnormal Doppler outcomes, such as absent or reversed end-diastolic flow, correlate with elevated rates of fetal demise and lower APGAR scores (assessed at five minutes), indicating compromised fetal circulation and a heightened risk of adverse outcomes. Conversely, normal or low-resistance Doppler findings are linked to improved fetal outcomes (Table 6). We found the highest fetal morbidity and mortality in high-risk pregnancies with reversed diastolic flow and/or those associated with the brain-sparing effect.

**Table 6:** Distribution based on Doppler findings of umbilical artery and its association with the fetal outcomes Data are presented as numbers. APGAR: Appearance, pulse, grimace, activity, and respiration; MCA: Middle cerebral artery

Doppler findings	No. of patients	Fetal outcomes		
		Low APGAR score	Fetal survival	Fetal demise
Normal	50	1	50	0
Reduced end-diastolic flow	39	15	37	2
Absent end-diastolic flow	24	11	18	6
Reversal of end-diastolic flow	7	7	1	6
Low resistance in MCA with brain-sparing effect	36	14	32	4

## Discussion

Fetal Doppler ultrasound has become an essential tool in managing high-risk pregnancies. By assessing blood flow in various fetal and maternal vessels, Doppler studies provide crucial information about the well-being of the foetus. This research study seeks to identify and further investigate the function of fetal Doppler in high-risk pregnancies and its effect on perinatal outcomes by comparing results with relevant studies. However, while the effect of abnormal Doppler on obstetric management has been observed, introducing this to regular clinical practice could reduce neonatal morbidity and mortality.

The average age of the 120 patients in our study was 26.76 years, ranging from 19 to 40 years. This is consistent with research by Kale et al., which found a mean age of 25.4 years, with a range of 18 to 37 years [3].

In our current study, 76 patients, or 63.33% of the total, were multigravidas. On the other hand, primigravidas made up 56.8% of the patients studied by Gaikwad et al. [4].

According to a National Survey among Indian women, the prevalence of high-risk pregnancies was 49.4%. Of these, 33% experienced a single high-risk pregnancy, while 16.4% had multiple high-risk pregnancies [5]. Our study observed a higher prevalence of hypertensive disorders and fetal growth issues. Specifically, PIH and preeclampsia together accounted for 28.33% of cases, followed by FGR at 18.34%, and preeclampsia associated with FGR at 15%. Other significant risk factors included GDM at 13.34%. These findings are consistent with Aparna and Suvarna, where approximately 90% of at-risk patients had preeclampsia, and 6% had GDM [6]. Similarly, Kumbar et al. reported 45% of their cases as PIH and 5% as GDM [7]. Based on our findings and those from the referenced studies, we can infer that the high prevalence of high-risk pregnancies, hypertensive disorders, and fetal growth issues at tertiary care centres is attributable to several factors. These centres frequently receive referrals for complicated cases, cater to populations with limited access to routine care, and often manage patients with advanced maternal age or pre-existing conditions. Furthermore, their superior diagnostic facilities and specialized care contribute to higher detection rates. Consequently, these centres tend to have a concentration of complex cases, making them well-suited for studying and managing high-risk pregnancies.

The UA usually displays a common spectral Doppler pattern called a "sawtooth" pattern in low-risk pregnancies, with the peak systolic velocity (PSV) at its highest position, the end-diastolic velocity (EDV) at its lowest point, and the time-averaged velocity (TAV) offering further information. The study, conducted at a single institution with a relatively small sample size, may not be widely applicable to other populations. The effectiveness of Doppler ultrasound is closely tied to the operator's skill and experience, and variations in technique and interpretation can affect the results and their reliability. There may also be selection bias, as the study only included women who delivered at the institution and those willing to participate, potentially omitting a segment of the high-risk population. Additionally, the study focused mainly on immediate maternal and neonatal outcomes, without long-term follow-up data for mothers and children, which limits understanding of the prolonged effects of Doppler findings. Although Doppler ultrasound provides valuable insights, its results should be considered in the broader clinical context to avoid over-reliance and potential misjudgements in clinical decisions. This pattern indicates continuous, unidirectional flow towards the placenta. Physiological changes take place in the latter half of the second trimester as a result of the placental villi's gradual maturation, the umbilical vessels' increased diameter and compliance, and the fetal heart rate and blood pressure. Flow velocity waveforms (FVWs) of pathological UAs show progressive alterations according to the severity of the disorder. While the PSV is constant, the waveform's EDV can change: it can become smaller (positive end-diastolic velocities (PEDVs)), completely vanish (absent end-diastolic velocities (AEDVs)), or reverse (reversed end-diastolic velocities (REDVs)). [8].

UA Doppler reflects the resistance in placental vascular flow, which correlates with intrauterine growth restriction and the systemic impacts of placental insufficiency. When UA blood flow becomes abnormal, Doppler assessments of systemic vessels, like the fetal MCA and ductus venosus, are necessary to better evaluate fetal status and aid in decision-making. The MCA is preferred for evaluating fetal cerebral circulation due to its clear identification [9]. In response to hypoxia, cerebral arteries dilate to maintain brain blood flow, resulting in a decreased systolic-to-diastolic ratio in the MCA, which indicates increased diastolic flow during chronic hypoxia. Doppler ultrasound detects this "brain-sparing effect" as a lower PI [10].

Evidence suggests that the UA Doppler is effective in assessing FGR and guiding pregnancy decisions. However, examining other vessels, such as the MCA or ductus venosus, can provide additional valuable information for decision-making.

In our study, a significant proportion of participants exhibited abnormal UA flow patterns: 32.5% showed reduced flow, 20% demonstrated absent flow, and 5.83% experienced flow reversal. Similarly, Kale et al. reported that 44.4% of their subjects had normal UA flow, while 18.9% showed reduced flow, 16.7% exhibited absent end-diastolic flow, and 20% had flow reversal in the UA [3].

In the current study, abnormal UA patterns were most prevalent in conditions such as FGR, PIH, preeclampsia, and FGR associated with preeclampsia. These high-risk groups accounted for 48.3% of the abnormal patterns in the study population. Varghese et al. compared absent versus reversed UA diastolic flow in high-risk pregnancies, with 57% having FGR and 38% having preeclampsia or eclampsia. Among those with reversed diastolic flow, 25% had preeclampsia or eclampsia, and 50% had FGR [11]. In the study by Gaikwad et al., abnormal UA RI and PI waveforms were found in 21.6% and 22.4% of patients, respectively. This differs from our study, which had a higher percentage of abnormal UA PIs. However, their findings on MCA Doppler studies were similar to ours, with 2.4% and 10.4% of patients showing abnormal MCA RI and PI, respectively [4].

Thirty women exhibited a reversed CPR in our study. Similarly, Karena et al. observed a reversal of the CPR in 34.6% of their research population, which is comparable to our findings [12].

In our study, the majority of patients (82.5%) underwent LSCS, while 16.67% had an NVD. Notably, a higher rate of caesarean sections was observed among patients with abnormal Doppler studies (60 out of 99). This reflects a significant predominance of caesarean deliveries in our study group, consistent with the global trend of increasing caesarean rates, especially in cases with abnormal Doppler findings. According to the National Family Health Survey (NFHS-4), 17% of live births in the Indian subcontinent during the five years preceding the survey were delivered by caesarean section. Of these, 45% were planned after the onset of labour pains. This represents a significant increase from the NFHS-3 data, which reported a C-section rate of 8.5%. The NFHS-4 data show that this rate has risen to 17.2%, reflecting a nearly 9% increase over a decade [13]. The high rate of caesarean sections, particularly among these patients, is influenced by several factors: the need to address fetal compromise, manage maternal health risks, respond to non-reassuring fetal status, and adhere to hospital protocols. Additionally, practices in defensive medicine and patient or family preferences contribute to the choice of caesarean delivery to ensure safer outcomes. Similarly, Messawa et al. also reported higher caesarean rates in patients with abnormal Doppler studies [14]. In contrast, Gaikwad et al.'s study found a higher rate of NVD at 56% [4].

Kale et al. found that 43.2% of the babies had low birth weight, compared to 50% noted in the current study [3]. Ali et al. compared foetuses with FGR that had normal and abnormal Doppler studies, finding a mean weight of 1,680 ± 259 grams for those with normal Doppler studies and 742 ± 126 grams for those with abnormal Doppler studies [15].

In our study, 63.33% of the infants were immediately admitted to the NICU after delivery, primarily due to low birth weight and preterm delivery, which accounted for 33.33%. Other reasons for NICU admission included hypoglycaemia evaluation, respiratory distress, and meconium-stained amniotic fluid. In Gaikwad et al.'s study, 88.8% of the 125 infants were admitted to the NICU for reasons such as low birth weight, asphyxia, and prematurity [4].

The APGAR score is assessed at one and five minutes after birth to gauge the baby's response to any medical interventions and to guide subsequent care. We reported that 39.16% of the infants had low APGAR scores. Tolu et al.'s study found that infants with abnormal UA Doppler studies had a twofold higher chance of having a low five-minute APGAR score compared to those with normal UA Doppler examinations [16].

We observed two intrauterine deaths and 14 infant deaths upon NICU admission, resulting in a neonatal mortality rate of 13.34%. Among patients with reversed end-diastolic flow, 85.7% experienced perinatal death.

Amin et al. [17], using colour Doppler ultrasonography in high-risk pregnancies, reported perinatal death and morbidity rates of 41.3% and 23.9%, respectively, with abnormal Doppler waveforms. Conversely, patients with normal Doppler waveforms had less morbidity (3.7%) and mortality rates (11.1%).

Fifteen infants (8.8%) died in the neonatal mortality study by Tolu et al. Of these, 4.5% were from the group with normal UA Doppler scans, and 24.3% were from the group with abnormal ones. According to this, newborns with abnormal Doppler investigations had a four-fold higher risk of neonatal death than those with normal UA Doppler examinations [16].

Limitations of the study

The study, conducted at a single institution with a relatively small sample size, may not be widely applicable to other populations. The effectiveness of Doppler ultrasound is closely tied to the operator's skill and experience, and variations in technique and interpretation can affect the results and their reliability. There may also be selection bias, as the study only included women who delivered at the institution and those willing to participate, potentially omitting a segment of the high-risk population. Additionally, the study focused mainly on immediate maternal and neonatal outcomes without long-term follow-up data for mothers and children, which limits understanding of the prolonged effects of Doppler findings. Although Doppler ultrasound provides valuable insights, its results should be considered in the broader clinical context to avoid over-reliance and potential misjudgements in clinical decisions.

## Conclusions

The study highlighted that fetal Doppler ultrasonography plays a crucial role in managing high-risk pregnancies and determining the optimal delivery timing, as observed in our study. By identifying foetuses at risk of placental dysfunction and growth retardation early in pregnancy, Doppler technology enables timely therapeutic interventions to promote fetal growth and development, thereby potentially improving long-term outcomes. The observed high rate of caesarean sections underscores the necessity for prompt intervention in high-risk cases with abnormal Doppler findings. In summary, antepartum fetal surveillance with obstetric Doppler ultrasound remains indispensable for improving perinatal outcomes in high-risk pregnancies.
